# Weaving relationships between “body techniques” and “self-care”

**DOI:** 10.3389/fsoc.2025.1554665

**Published:** 2025-03-28

**Authors:** Maria Isabel Brandão de Souza Mendes

**Affiliations:** Research Group on Body, Health and Playfulness – SALUD, Postgraduate Program in Physical Education, Department of Physical Education, Federal University of Rio Grande do Norte, Natal, Brazil

**Keywords:** body, self-care, sociology, philosophy, epistemology, physical education

## Abstract

Epistemological questioning is necessary for any knowledge area because it brings up reflections on its foundations. The objective of this article is to understand the relationships between studies on “body techniques” and “self-care.” This is a bibliographical study on the relationships between “self-care” and “body techniques” based on the studies of Foucault and Mauss and based on updated articles on these concepts. We situate the context in which the notion of body techniques and then the notion of self-care emerge. We subsequently correlate the classification of body techniques developed by Marcel Mauss and establish relationships with the possibilities of self-care. Next, we identify that many body techniques are part of the practices of the regime, which have existed since the Socratic-Platonic moment in our Western society. We point out that Physical Education/Sports Science has a crucial importance in teaching self-care. We emphasize that professionals in this knowledge area can open spaces for reflection on body techniques which contribute to self-care and do not dictate norms, as subjects are capable of making choices based on their learning.

## 1 Introduction

Epistemological research is important for any knowledge field, as it questions theories, concepts, nature and limits of knowledge. These studies are reflective activities on science and its foundations (Soler, 2019). In this article, we focus on the humanities, especially Sociology and Philosophy, with a view to contributing to Physical Education/Sports Science. Our focus is on body studies and we will discuss two notions, namely: “body techniques” and “self-care.”

In the book “The Sociology of the Body,” Le Breton (2024) demonstrates that the study on the notion of “body techniques” was presented by Marcel Mauss in 1934 to the French Psychological Society.

Body techniques refer to “the ways in which men from society to society, in a traditional way, know how to use their bodies,” meaning they are related to the uses of the body in different societies, as highlighted by Mauss (2003, 401). In this way, we associate body techniques with the uses we make of our bodies according to the society in which we are inserted.

By highlighting this notion, David Le Breton shows that body techniques are “gestures codified with a view to practical or symbolic effectiveness, they are modalities of action, sequences of gestures, muscular synchronies that follow one another in the search for a precise purpose” (Le Breton, 2024, 45).

When we turn to Physical Education/Sports Science, we identify that this knowledge area has worked with “body techniques” in several studies, but especially in pedagogical studies, such as those by Rodrigues (2000); Alkemeyer (2002); Daolio (2005); Lüdorf (2009); Shilling (2010); Andersson et al. (2013); Cavalcante and Potiguar Junior (2019); Santos (2020) and Scaglia et al. (2020), among others. However, studies on “body techniques” focused on epistemological activity are still scarce.

In the book “The Sociology of the Body,” Le Breton (2007) demonstrates that studies on the notion of “self-care” bring an epistemological change in Michel Foucault’s studies, where the author shifts his gaze from the repressive hypothesis present in biopolitical studies to a look at subjectivities.

“Care of the self” refers to attention to the body that varies in different socio-historical scenarios, as presented by Foucault (1984, 1985, 2006, 2018) in several works, such as History of Sexuality related to the uses of pleasures and care of the self, as well as in Hermeneutics of the subject.

The concept of “self-care” emerges in the third moment of Michel Foucault’s academic trajectory and is related to genealogy of the subject. In this sense, it is important to highlight that there is a broad field of research to be developed on “self-care,” mainly in the area of Physical Education/Sports Sciences, since researchers in this area still emphasize Foucault’s studies related to biopolitics.

As Mendes and Gleyse (2014, p. 509) point out:

One of the hypotheses for this lack of discussions on self-care may be the fact that studies in Physical Education that seek contributions in the works of Michel Foucault have emphasized other concepts addressed by this French philosopher, such as the concept of disciplinarization, biopower and biopolitics, for example.

In this sense, we recognize the importance for Physical Education/Sports Sciences of studies at both national and international levels on the body with a focus on the concept of “self-care,” as it reconnects knowledge and practices. At this point, we will focus on the relationships between “body techniques” and “self-care,” because there is no epistemological study that establishes relationships between these themes based on the studies of Marcel Mauss and Michel Foucault.

Furthermore, we currently live in a context in which humanity often fails to explore its bodies through movement. We increasingly sit in front of our cell phones, computers and televisions. We also spend a lot of time sitting in our cars and in our offices. As a result, a sedentary lifestyle is a public health concern, as Le Breton (2020) points out, and we neglect body techniques that could contribute to self-care.

In order to contribute to this debate, our study questions are:

- How can we relate the studies on “body techniques” by Marcel Mauss and “self-care” by Michel Foucault?
- What “body techniques” can contribute to “self-care”?

Thus, our objective herein is to understand the relationships between studies on “body techniques” and “self-care.”

This study may contribute to the dialogue between sociological and philosophical knowledge and expand knowledge production in Physical Education/Sports Sciences related to epistemological activity. In addition, it may contribute to recognizing and appreciating sociophilosophical studies on the body and self-care with the aim of denaturalizing them, as well as pointing out elements for public health and leisure policies.

## 2 Materials and methods

As we are inserted in a hermeneutic paradigm, we will focus on the interpretation of texts in the sense exposed by Loland and Mc Namee (2017). This is bibliographic research on the relationships between “self-care” and “body techniques” based on the studies of Foucault (1984, 1985, 2006, 2018) and Mauss (2003) and based on updated articles on these concepts.

Bibliographic research “is developed from material already prepared, consisting mainly of books and scientific articles. […] Part of the exploratory studies can be defined as bibliographic research” (Gil, 2008, p. 50).

This type of research follows some steps as highlighted by Gil (2008). The first is formulating the problem according to what is intended to be researched. To do so, we emphasize that our problem was how to relate the studies on “body techniques” by Marcel Mauss and “self-care” by Michel Foucault. Another problem presented was knowing how “body techniques” can contribute to “self-care.” Then, a work plan is constructed with items directed into sections to direct the research. Our work plan was built with the idea of highlighting the main points of the texts to be analyzed on “body techniques” and “self-care,” among which we highlight: the meaning of these notions and the highlights of the texts mentioned by the authors. After constructing the work plan, the sources that will contribute with the answers capable of resolving the problem are identified. The sources were chosen because they are where the authors discuss these notions. “Body techniques” are discussed in the book “Sociology and Anthropology” by Marcel Mauss, and “self-care” is discussed in the books: “History of sexuality 2: the use of pleasures”; “History of sexuality 3: the care of the self”; “Hermeneutics of the subject”; and “History of sexuality 4: confessions of the flesh.”

Immediately after identifying the sources, we try to find out where they are. After having the chosen sources, we first do an exploratory reading of the selected material. Once we have defined the texts to be researched, we move on to selective reading, which is an in-depth reading of the passages that are most interesting. Next, we do an analytical reading with the objective to organize and synthesize information from the sources. We try to identify the main ideas of the text and order them to make the synthesis. Finally, we do an interpretative reading, which sometimes occurs together with the analytical reading (Gil, 2008). After reading the sources chosen for this study, it is at this point that we identify the meanings of the notions of “body techniques” and “self-care,” in addition to highlighting the main points addressed by the respective authors in the books.

For Sousa et al. (2021, p. 66), bibliographic research:

Is based on the study of previously published theory, so it is essential that the researcher appropriates the domain of reading knowledge and systematizes all the material that is being analyzed. When carrying out bibliographic research, the researcher has to read, reflect and write about what they have studied, dedicate themselves to the study to reconstruct the theory and improve the theoretical foundations. It is essential that the researcher organizes the selected works that collaborate in constructing the research in the form of cards.

In order to organize the construction of the research, it is important that all relevant points arising from the material read are noted in documentation, bibliographic and note cards. The bibliographic cards record the bibliographic references, a summary and a critical assessment of each work. The note card serves to record the ideas acquired from each text read. Next, logical construction of the work begins, organizing the ideas to meet the research objective. Once the definitive study plan is in hand, the writing of the work begins (Gil, 2008). In this context, after creating files with the identified content, we began writing and divided the article into three parts. The first is called “In the intricacies of body techniques”; the second is “Scenarios about self-care”; and the third is “From the uses of the body to practices of the self.” The study conclusion immediately follows.

## 3 In the intricacies of body techniques

Marcel Mauss was Émile Durkheim’s nephew and was influenced by his uncle’s Sociology, collaborating with it, but above all knowing how to free himself from it, with a view to moving away from the “old references to biologism, naturalism and social evolutionism” (Le Breton, 2021, p. 7).

Even before Marcel Mauss presented the notion of body techniques to the Psychological Society in 1934, he had already previously addressed this notion in his teachings at the Institute of Ethnology and at the “Collège de France” after the war (Vuillermet, 2023).

According to Mauss (2003), there was discomfort with social facts such as walking and swimming being studied under the nomenclature of “various.”

I knew perfectly well that walking, swimming, for example, that things like that were specific to certain societies; that the Polynesians do not swim like us, that my generation did not swim like the current generation. But what social phenomena were these? They were “diverse” social phenomena, and, as this heading is a horror, I thought about this “diverse” several times, at least every time I was forced to speak about it, from time to time (Mauss, 2003, p. 401–402).

This discomfort led him to create the notion of “body techniques” through various observations and his previous studies, first on swimming. Later, the author began to observe the way French and English troops dug their horses, as well as their way of marching. With these and other observations, Mauss (2003) identified that every body attitude varies from society to society, as each one has its own habits. He tells us that his revelation regarding “body techniques” came when he was hospitalized in New York. He noticed that he had seen young women walk like nurses and was able to identify that it was in the movies. In addition, Marcel Mauss began to observe French women who also walked this way, demonstrating the dissemination of this gesture through cinema, social influences and a certain form of education in walking.

The habits that Mauss (2003, p. 404) refers to vary according to individuals and their imitations, but mainly “with societies, education, conveniences and fashions, prestige.” Given this context, the author shows the interrelation between biological, psychological and social aspects in what he calls “total man,” meaning a human being who cannot be understood in just one of these aspects, as they are integrated, overcoming the naturalization of gestures.

These body techniques are learned and reproduced with the appropriate refinements and style of each human being. These techniques related to gestures are rooted in the evidence of the relationship to the world (Le Breton, 2021).

When talking about “body techniques,” it is worth noting that Marcel Mauss relies on the notion of “incorporation,” or “on the somatic integration – by the body – of a range of postures and behaviors” (Vuillermet 2023, p. 29). Considered to be of a social nature, these behaviors are traditional and effective, as well as arbitrary and necessary, and they construct each person’s identities (Vuillermet, 2023).

Body techniques are not a simple mechanical application, but at every moment they are a dynamic reformulation of internalized propositions. Furthermore, these techniques are always sensory techniques. As Le Breton (2021, p. 31) points out, body techniques are “a way of feeling forces, textures, colors, sounds… and reacting to them immediately.”

Mauss (2003) developed principles for classifying body techniques, varying by sex and age, but also in relation to performance and transmission forms. In addition, the author carried out a biographical enumeration of body techniques. In this context, he reports on birth and obstetrics techniques, childhood, adolescence and adulthood techniques, activity techniques, meaning movement, body care techniques, consumption and reproduction techniques and medication techniques.

Without a doubt, his classification work is of utmost importance for socio-anthropological studies. However, we identified the beginning of a classification in his study which is still open for future studies. For Le Breton (2021), Mauss did not intend to propose a precise and exhaustive research program. More than that, Marcel Mauss encouraged us not to ignore the details, the tiny and seemingly insignificant elements of human behavior, which also have meaning and attest to the fact that they are part of a broader social symbolism, establishing a broad research field.

## 4 Scenarios about self-care

Michel Foucault was a professor at the Collège de France from 1971 until his death in 1984, in the chair called History of Systems of Thought. It was on January 6, 1982 that he began to teach classes on “care of the self” in a course that became the book “The Hermeneutics of the Subject.” As the author himself says, “care of the self” already existed before he had studied the socio-historical periods.

Therefore, the philosophical reflection on “self-care” linked to Western society emerged in the Socratic-Platonic period. “Self-care” means taking care of oneself and being concerned about oneself and is related to self-knowledge. Self-care was aimed at learning to care for others in order to be able to govern cities (Foucault, 2006).

It is interesting to note that taking care of oneself is always related to taking care of others, whether through a guide, a teacher or even a friend. It is by no means an exercise in solitude. It is therefore a social practice. Let us observe the role of Socrates when he spoke to young people about the need for them to take care of themselves. For Socrates:

In the activity that consists of inciting others to take care of themselves, he plays, in relation to his fellow citizens, the role of the one who awakens.” The care of the self will therefore be considered as the moment of the first awakening. It is situated exactly at the moment when the eyes open, when one emerges from sleep and reaches the first light (Foucault, 2006, p. 11).

In these interactions of Socrates to awaken young people on the streets, we identify that self-care is linked to a subject inserted in a context in which he weaves social relationships throughout his life.

According to Foucault (1985), self-care reached the idea that taking care of oneself is an imperative and a way of acting, a way of behaving. It suggests ways of living developed in procedures, actions and recipes that were the basis of reflections and teachings. Self-care “thus constitutes a social practice, giving rise to inter-individual relations, exchanges and communications and even institutions; it provided, finally, a certain mode of knowledge and the elaboration of knowledge” (Foucault, 1985, p. 50).

When we move to the 1st and 2nd centuries AD, which is the golden age of self-care, we identify that self-care becomes associated with the arts of living. There is a chronological change and self-care is for all individuals, at all times and without status conditions. The objective to be achieved becomes the end in itself and no longer the city, as previously, in the Socratic-Platonic period. Furthermore, the main form becomes associated with a practice of the self and no longer with self-knowledge (Foucault, 2006).

In the years III and IV AD, considered the Christian moment, self-care is related to self-renunciation and not to returning to oneself, as in Greco-Roman Antiquity. The proposal is sacrifice, the renunciation of oneself based on a word spoken by another. The Christian confession begins to guide the subjects (Foucault, 2012a, 2012b, 2018).

In the Christian period, III and IV AD, self-care is linked to the renunciation of all earthly connections, to everything that can be recognized as attachment to the earthly self. From Antiquity to Christianity there is a transformation of a morality that was in its essence the search for a personal ethic to a morality as obedience to a system of rules (Mendes and Gleyse, 2014, p. 515).

From Antiquity to Christianity, the way in which “self-care” developed changed. From the construction of the subject permeated by choices, it transformed into a construction guided by religious dictates.

But why did Michel Foucault need to look to Greco-Roman Antiquity to conduct his studies? Precisely because this philosopher went to the past to reflect on the present. His intention was to debate the relationship between subjectivity and truth. And it was in Greco-Roman Antiquity that the aforementioned author found greater importance given to the practices of the self and the autonomy of subjects, greater than in socio-historical scenarios guided by religious, pedagogical and medical institutions, as highlighted in an interview given to the International Journal of Philosophy in 1984 (Foucault, 2010).

In Greco-Roman antiquity, these practices of the self associated with “self-care” are practices of self-formation of the subject, a practice considered ascetic, but with another meaning given by Foucault (2010, p. 265): “not the sense of a morality of renunciation, but that of an exercise of the self upon oneself through which one seeks to elaborate, transform oneself and achieve a certain way of being.” In this context, Michel Foucault does not speak of a general and universal subject. In weaving his discussion on “self-care” he traces a historical constitution of different forms of subject associated with games of truth.

## 5 From the uses of the body to practices of the self

The uses we make of our bodies, meaning the body techniques we use, are individual and collective, since we are part of a society. Thinking from this perspective today, what body techniques help us take care of ourselves? Can the body techniques that contribute to self-care be considered self-practices? Based on these questions that we have now raised, we will draw connections between these notions.

First, we will highlight the body techniques, using their classifications established by Marcel Mauss (2003) and reflecting on how they can contribute to self-care.

Let us look at the division of body techniques between the sexes that Mauss (2003) talks about, highlighting the way women and men close their fists. Women close their fists with their thumbs inwards and men with their thumbs outwards. Given this debate, the following question arises: Do women, men, non-binary people, among others, take care of themselves in the same way? In this sense, a range of research possibilities opens up to investigate the differences between these subjects in body techniques for self-care.

Regarding the variation in body techniques with age, we ask how children take care of themselves? We think that children receive care from their parents and as they grow, they learn to take care of themselves. Children learn to feed themselves, for example. In adolescence, autonomy is acquired based on what has been learned up to that point in relation to self-care. Adults also choose their care techniques according to their learning. According to Le Breton (2021, p. 32):

The acquisition of body techniques by actors comes from a generally very formalized education, implemented intentionally by those around the child (or the adult who seeks to appropriate another use of the things of the world). This learning continues, sometimes adjusting throughout life, for example regarding the emergence of new techniques or their evolution.

Mauss (2003) also describes sleeping techniques which vary from place to place and can contribute to self-care. Sleeping in a hammock or bed, sleeping on the floor, sleeping on mats, and even sleeping standing up like the Masaï, a group of Africans. In addition, sleeping more or less than 8 h a day, or sleeping with your cell phone next to your body, all change according to society and personal preferences, and may or may not contribute to self-care.

There are the rest techniques mentioned by Mauss (2003) that can also contribute to self-care. However, these techniques are not universal, as there are people who rest sitting on chairs, others on mats, as in the East. As the author highlights:

Some societies rest in unusual positions. Thus, the whole of Nilotic Africa and part of the Chad region, as far as Tanganyika, are populated by men who, in the fields, stand like wading birds to rest. Some can stand on one foot without help, others support themselves with a stick (Mauss, 2003, p. 415).

Other techniques cited by Mauss (2003) are movement techniques. There are societies where the climate is conducive to skiing in the snow, such as in the Swiss Alps; other societies are more focused on running, whether with shoes or barefoot; and there are even log races among Brazilian indigenous people. Thus, the ways in which the body moves change according to the socio-historical period and can contribute to self-care.

Regarding body care techniques, Mauss3 mentions hygiene techniques, in addition to the use of the body when bathing, whether in bathtubs, showers or even in streams, all of which can contribute to self-care. In addition, we also highlight the use of cosmetics to moisturize the skin or for makeup with industrialized or even natural products, as in the case of indigenous people. Beautifying oneself can also be considered self-care. As Le Breton et al. (2013, p. 219) highlights:

If beauty is a form of care, then it is no longer a question of questionable narcissism; it is also a concern for health. Beautifying the skin is a way of healing it, regenerating it, and restoring its youth. And doing nothing is an unfortunate abstention for your health. Beauty care is now a happy version of medicine, far removed from illness, and instead aims to increase well-being and prolong the taste for life. Concern for care becomes self-care.

There are also consumption techniques, such as eating or drinking, which vary according to the preferences of societies and can contribute to self-care. Eating sitting at the table or sitting on the floor, like Oriental cultures. Eating slowly, savoring food, eating with your hands or with a knife and fork, among other aspects. According to Lipovetsky (2021), today people seek to eat organically and locally. Eating healthy and natural, everything that constitutes an alternative to industrial food.

Regarding reproduction techniques, sexual acts can also contribute to self-care. “Touching for sex, mixing of breaths, kisses, etc.” (Mauss, 2003, 419). Here we associate this with the uses of sexual pleasures cited by Foucault (1985).

There are also medication techniques, such as massages (2003). These can be relaxation, shiatsu, Thai, Ayurvedic, among others, and can help with self-care. According to Vigarello (2016), projects involving self-action emerged in the West at the beginning of the 20th century, such as intervention methods (relaxation, self-suggestion, dance, rhythmic exercises that aid in concentration and expression practices).

All the body techniques mentioned above were already part of the self-care practices associated with diet, or regimen, since the Socratic Platonic period: physical exercise, ways of eating and drinking, sleep and sexual relations. In this period, the regimen was considered a health concern and at the same time a way of conducting one’s existence (Foucault, 1984).

We also have in Marcel Mauss’ study the body techniques in relation to performance, but how can we associate this with self-care? According to Mauss (2003), these techniques refer to skills in something. In this sense, we can relate the body techniques in relation to performance to the skills of taking care of oneself.

It is also important to raise some questions about how different body techniques are associated with specific symbolic meanings in different cultural contexts. For example, we can see that Tango emerged as a popular dance in Argentina, and in order to be disseminated in Europe during the First World War, there was a need for a change in its gestures, such as fixing steps, distancing itself from the low and dramatic body of the Tango of its origin. As Nóbrega (2003, p. 136) highlights, the Tango danced in Argentina:

Is another gesture, another look: direct and passionate. A popular dance, in the sense of social belonging and resistance in maintaining an identity of a Latin America that has not allowed itself to be dominated, at least in its dance, by Eurocentric culture. This is the body of Latin American Tango, strong and fragile, determined, dramatic, driven by passion and not by bourgeois or aristocratic etiquette.

Given this context, we reinforce the idea that different body techniques express different meanings according to the culture in which they are inserted, and these symbols can influence the way people perceive themselves and others.

Furthermore, another interesting approach is that in the same way that there is an education in body techniques (Mauss, 2003), we highlight that there is an education in the uses of the body for self-care. Teaching these techniques is passed down from generation to generation through the family, or at school, or even through the influence of friends and the media.

It is interesting to identify that the uses of the body for self-care can be recognized as practices of the self, when subjects construct themselves through their care and the care of others who interact with them. For Foucault (1985, p. 62), the practice of the self:

Implies that the subject constitutes himself in relation to himself, not as a simple imperfect, ignorant individual who needs to be corrected, formed and instructed, but rather as an individual who suffers from certain illnesses and who must have them taken care of, either by himself or by someone who has the competence to do so.

In this sense, body techniques which contribute to self-care are both personal and social and contribute to the understanding that individuals can appropriate different uses of the body as they grow and mature in their lives according to their learning and personal tastes.

## 6 Conclusion

Understanding the relationships between studies on “body techniques” and “self-care” can broaden epistemological activity in Physical Education/Sports Sciences, both in Brazil and internationally. The dialog between Sociology and Philosophy can expand the hermeneutic paradigm through interdisciplinarity.

First, we situate the context in which the notion of body techniques and then the notion of self-care emerge. Subsequently, we correlate the classification of body techniques developed by Marcel Mauss and establish relationships with the possibilities of self-care.

Despite the notion of body technique having been presented in 1934 to the Psychological Society, it continues to be very important for current studies, as it demonstrates that gestures are constructed and reconstructed according to the socio-historical contexts in which we are inserted. Body techniques, as presented by Marcel Mauss, denaturalize the issue of gestures, as biological aspects are intertwined with psychological and social aspects.

The notion of self-care appears in Western society in the Socratic Platonic period, where it was linked to the idea of taking care of oneself and related to self-knowledge with a view to being able to govern cities. Self-care in the 1st and 2nd centuries AD was associated with the arts of living and in the Christian period with self-renunciation. In this sense, we can observe how self-care varies according to each socio-historical context. By relating the classification of body techniques to the possibilities of self-care, we raise contemporary questions such as: do women, men, non-binary people, among others, have the same way of doing things? These questions cannot be immediately answered by this study, but that may instigate other researchers to develop new studies.

We then identify that many body techniques are part of the regime practices that have existed since the Socratic-Platonic period in our Western society. Furthermore, we show that the uses of the body for self-care are also forms of self-practices, when subjects construct themselves through their care and the care of others.

In this context, we point out that Physical Education/Sports Science is crucially important in teaching self-care. We emphasize that professionals in this knowledge area can open spaces for reflection on body techniques that contribute to self-care and do not dictate norms, as individuals are capable of making choices based on their learning.

We therefore point out the need for Physical Education/Sports Science professionals to collaborate with public health and leisure policies based on the uses of the body for self-care and for the care of others. These public policies should not only consider the human body in its complexity and only focus on organic aspects, but also be capable of considering cultural, social and historical aspects.

Finally, we point out the need for further studies on these topics, whether theoretical or field studies, which may provide further analyses or further exploration of some of the questions raised in this study. Some suggestions can be made, such as identifying ruptures and continuities in the body techniques currently used for self-care. Another suggestion would be to analyze educational programs to identify how they can promote greater awareness and understanding body techniques and their relationship to self-care.

## Data Availability

The original contributions presented in the study are included in the article/supplementary material, further inquiries can be directed to the corresponding author.
